# Turning over New Leaves

**Published:** 1890-07-05

**Authors:** 


### Turning Over New Leaves.

**Browning's Message.***

It may be that it is yet too soon to say what is Browning's message to his time. He still stands too near us; and, our minds overshadowed by his large personality, we are unable to see the true proportions of either the man or his work, and so dilate chiefly on those aspects of both that stand intellectually closest to ourselves. This is what the critics have been doing ever since Browning's death—both those who have praised and those who have blamed. We are told on the one hand that Browning, if he is unappreciated to-day, will be the poet of to-morrow, or at least of the day after; a prophecy regarding which silence is discreet, for after all we don't know what posterity will do. On the other, we are told that Browning's work is careless, unpolished, inartistic, and that they of the day after to-morrow will refuse to regard him as a poet at all. And again it is wise to keep silence. The men and women of the generation after ours will form their own opinion. Likely enough it will be a correct one, but we cannot foretell its nature. But, since it is our instinct to talk of those whom we have honoured, or whom we have heard praised, we give our own versions of "Browning's Message to His Time," meaning, as a rule, his influence on our individual brain and soul.

Browning's message, be it weighty or trivial, is to the brain and soul. He has little to say to that indefinable something, that mingling of the sensuous and the spiritual, which we call the artistic faculty. Nay, he outrages it wilfully—wantonly. He could sing once, though only by fits and starts; having the passion of the bard, but not the conscience of the artist, who labours at his work till the very cadence of the words echoes the measure of the thought. He defies the conventions, the very decencies of the art he professes: he perpetrated the awful rhyme, "Italy—spit ally." It has been held as a virtue that to him the matter is all, and the form naught; but what right has a man to call himself a poet if, though the stuff he brings be brocaded with the richest flowers of thought, he refuses to shape it into a fitting garment for the Muse? Then he is self-conscious and over-clever. He will play you any metrical tricks you choose; there is no form so difficult that he cannot rhyme you in it, though often words and ideas are packed to over-crowding within the cramping limits of the verse. You perceive the dexterity of it all; perhaps admire it, but it is not art; *ars est celare artem.*

If, then, poetry has any technical requirements beyond rhyme and scansion, if beauty of phrase and rhythmic flow of sound be integral parts of it—but on these points critics differ wondrously—Browning as a poet has not much to say. He has written some poems, but the sixteen volumes of his published works are not by any means all poetry. So much for Browning the poet, but with Browning the thinker, the moralist, the preacher, it is another matter, and Dr. Berdoe in dealing with "Browning's Message to his Time" does wisely to dwell on his science, his philosophy, and his religion. He had all three in no small measure, and there are few of his professed admirers who will not do well to note and take to heart the fact that a man who, whatever his defects on the æsthetic side, had a powerful and acute intellect, and who was widely and deeply read in the science of which our own day is so proud, as well as the philosophy in which past ages gloried, was not merely a religious man—*rococo* and absurd as religion seems to every sage of twenty-one!—but was a firm believer in the gospel of Jesus Christ. We have, however erroneously, got so much into the habit of associating intellect with agnosticism that this fact is worth noting and dwelling on. Browning's mind was essentially of a religious type; it had the completeness of true religion. He had the firmest regard for the body and its rights; he perceived "the value and significance of flesh" as clearly as any of his contemporaries. Whitman "sending his barbaric yawp over the roofs of the world" in praise of animal strength, Swinburne hymning the ecstasy of passion in words of swooning sweetness, do absolutely less justice to humanity than Browning. For he always takes the body *plus* the soul, which tends to completeness of view, and fuller knowledge even of flesh and blood. The brain and the spirit dominate him, and while he has a very stinging word for the "scientist" who vows

> "At the expense
> Of half an hour and eighteenpence,
> How brain secretes dog's soul we'll know,"

he has in him something of the moral vivisector. Any act of mortal man or woman, any display of love or hate, heroism or crime, so catches him that he cannot rest till he has evolved the metaphysic of it—nay, till he has pictured every possible explanation of the act. Who but he could have preached a sermon the length of "Fifine at the Fair," from a girl dancing on the tightrope—a sermon that touches on almost every spring of emotion in a woman's nature, with minor excursions into the domain of things in general? Who but he could analyse the motives, the contrasting views of martyr and murderer, of passion-tossed priest and saintly pope, as is done in "The Ring and the Book"? A wonderful piece of work that! We all admit it, as Carlyle did; some of us, however, adding, with Carlyle, that it is made out of a story "that only wanted forgetting."

There can be no doubt that, more than his alleged obscurity, this wilfulness in the choice of subject has hindered Browning's popularity. And rightly so. It is a wholesome instinct that makes us desire to dwell on noble deeds and thoughts, on faithful love, on loyalty, on heroism. We do not want to stay too long in the company of "Mr. Sludge, the Medium;" we can't—being only average members of the commonplace British public—altogether despise the weakness that kept the characters in "The Statue and the Bust" from sin. Our Browning, though we love him, is a shade too catholic for our tastes, too ingenious in suggesting all the motives that may induce to virtue or to sin.

He is too clever; that is his misfortune. He dazzles and bewilders us by the multiplicity of his fancies, the dexterity with which he shows the goodness of the erring, the fame of the forgotten. Then he lacks the divine gift of the artist—self-restraint, self-effacement. Through lyric, epic, drama, there is always one clear defined character — Robert Browning, making his puppets dance, and showing you how he does it. And this in spite of the fact that of all virtues none appealed to him more than the self-sacrifice of the man who loses himself in his work. Who are his heroes? Paracelsus, the vilified reformer of medicine, whose name was made a synonym for vain boasting, even while the world was profiting by his discoveries; the "Pictor Ignotus," who quietly accepts oblivion as his doom; the people "of importance in their day" who are forgotten in ours, though some of the effect of their work survives; on these and their like he loves to dwell. Perhaps there is a fitness in the choice; perhaps Browning himself may yet be one of these. It is possible—forgive us, O ardent Browningite, when we say that it is possible that, as a poet, Browning may not survive! He may, to coming generations, be one of the great unread; but as a moral force of the nineteenth century he cannot be ignored. His life has taught patience and courage under contemptuous neglect; in his work he has defended the weak, justified the

* "Browning's Message to His Time: His Religion, Philosophy, and Science." By Edward Berdoe, M.R.C.S., L.R.C.P. (Edin.) (London: Swan Sonnenschein and Co.)

misunderstood, excused the erring. Allow for all we can of accident in his fame, find every fault we can with his methods and his work, there still remains the fact that he has bequeathed to his readers a series of lessons in courage, industry, and tolerance, which form a sufficient and precious "Message to his Time."

**In Flight from the East Wind.***

Mr. Reynolds Ball's little volume is just what it professes to be, a practical handbook to the many nooks around the central sea where summer dwells in the midst of our winter. It does not profess to be a complete guide-book to any one place, but it states clearly the advantages and disadvantages of climate, society, and expense, of the health resorts of the Riviera, Spain, Sicily, Corsica, Malta, and Northern Africa. To anyone who wants to go abroad, but has no experience to guide him in the choice of a place, it is of the greatest value to have a volume like this to tell him not only of winds and sunshine, but of the charges at hotels and boarding-houses, the chances of amusement, the names of English doctors and bankers, and all those details about which the intending traveller, especially if he be an invalid, feels most anxious. For preliminary guidance, he could not do better than consult this little book.

* "Mediterranean Winter Resorts: A Practical Handbook to the Principal Health and Pleasure Resorts on the Shores of the Mediterranean." By E. A. Reynolds Ball. (London: L. Upcott Gill, 170, Strand.)

**A Practical Cookery Book.***

This little book is one of the most lucid and practical of its kind. The recipes are not numerous, but they are mostly typical, and can be varied almost indefinitely by anyone possessed of a little thought and ingenuity. The instructions as to cleaning vegetables and fish and preparing meat for cooking, are a distinctly valuable part of the book; and the diagrams showing the different modes of cutting up oxen and sheep in England and Scotland should be of use to young housekeepers. Not less valuable is the warning that there are some things which cannot be taught by any book, but must be acquired by practice in the kitchen. This is a matter which the purchasers of cookery books often forget, and thence arise unappetising dishes, bad temper, and indigestion.

* Middle Class Cookery Book, compiled for the Manchester School of Domestic Economy. (Macmillan and Co.)

